# Supplementary material for: Development of daily downscaled, bias-corrected CMIP6 climate datasets for estimating reference evapotranspiration (ETo) in South Asia
Source: Sci Data. 2025 Nov 28;12:1879. doi: 10.1038/s41597-025-06149-4 (PMC12663131; doi:10.1038/s41597-025-06149-4)
Supplement: Supplementary file 1 — Development of Daily Downscaled, Bias-corrected CMIP6 climate Datasets for estimating ETo in South Asia [file 41597_2025_6149_MOESM1_ESM.docx]

**Supplemental Information**

**Development of Daily Downscaled, Bias-corrected CMIP6 climate Datasets for estimating ET_0_ in South Asia**

Aniruddha Saha^1^, Manoj Kumar Jain^1*^, Pranita Joshi^1^, Subhankar Das^2,3^, Naimesh Singh Rawat^1^

^1^Department of Hydrology, Indian Institute of Technology Roorkee, Roorkee, India.

^2^Postdoctoral Researcher, Department of Soil and Water Systems, University of Idaho, Moscow, ID, USA

^3^Postdoctoral Researcher, Moscow Forestry Sciences Laboratory, USDA Forest Service-Rocky Mountain Research Station, Moscow, ID, USA

*Corresponding author Email: [manoj.jain@hy.iitr.ac.in](mailto:manoj.jain@hy.iitr.ac.in)^*^

**Table of contents**

| **Figure/Table** | **Page No.** |
| --- | --- |
| TableS1 | 2 |
| Fig. S1 | 2 |
| Fig. S2 | 4 |
| Fig. S3 | 6 |
| Fig. S4 | 8 |
| Fig. S5 | 10 |
| TableS2 | 11 |
| TableS3 | 12 |
| TableS4 | 13 |
| TableS5 | 14 |
| TableS6 | 15 |

Table S1 : Percentage of arid regions over South Asia under various climate change scenario

| **Climatic Scenario** | **% Area** |
| --- | --- |
| Observed (1991-2020) | 34.47 |
| SSP126 (2071-2099) | 32.83 |
| SSP245 (2071-2099) | 33.08 |
| SSP370 (2071-2099) | 33.16 |
| SSP585 (2071-2099) | 33.14 |





Figure S1 : Climatology for each climate variable before (original, blue line) and after (bias-corrected, dashed blue line) bias-correction against the ERA-5 reference climatology (red line) for each CMIP6 GCM model over the historical period (1960-2014). The climatology of each variable is as follows: monthly mean of mean daily temperature (i - xii), monthly mean of mean daily solar radiation (xiii - xxiv), monthly mean of mean daily wind speed (xxv - xxxvi), monthly mean of mean daily relative humidity (xxxvii - xlviii).

#
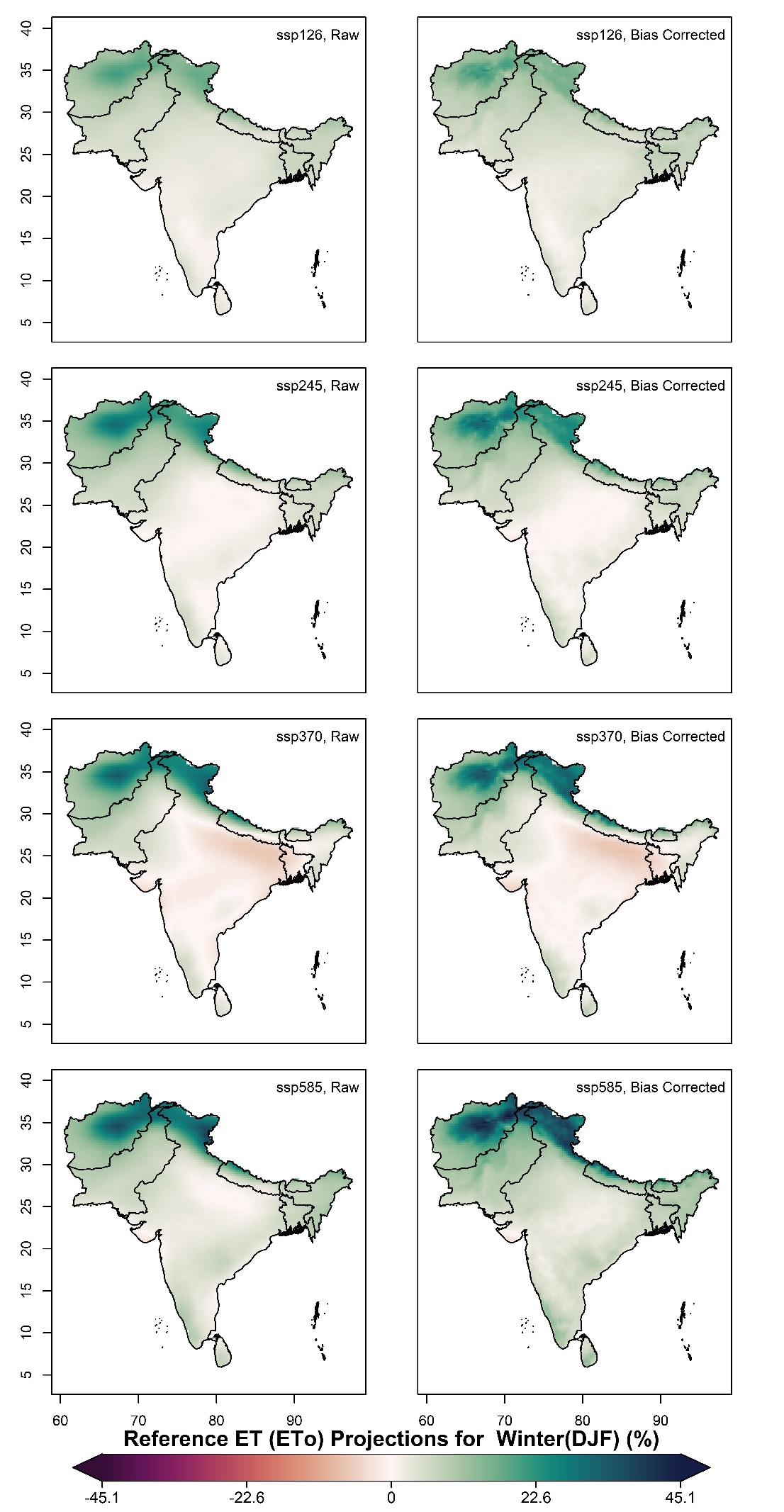


**Figure S 2 :** Multimodel ensemble median projected change in winter(DJF) mean ETo (%) for the far-future (2066-2100) with respect to the historical period (1960-2014) in original simulations (a, c, e & g) and bias-adjusted simulations (b, d, f & h).


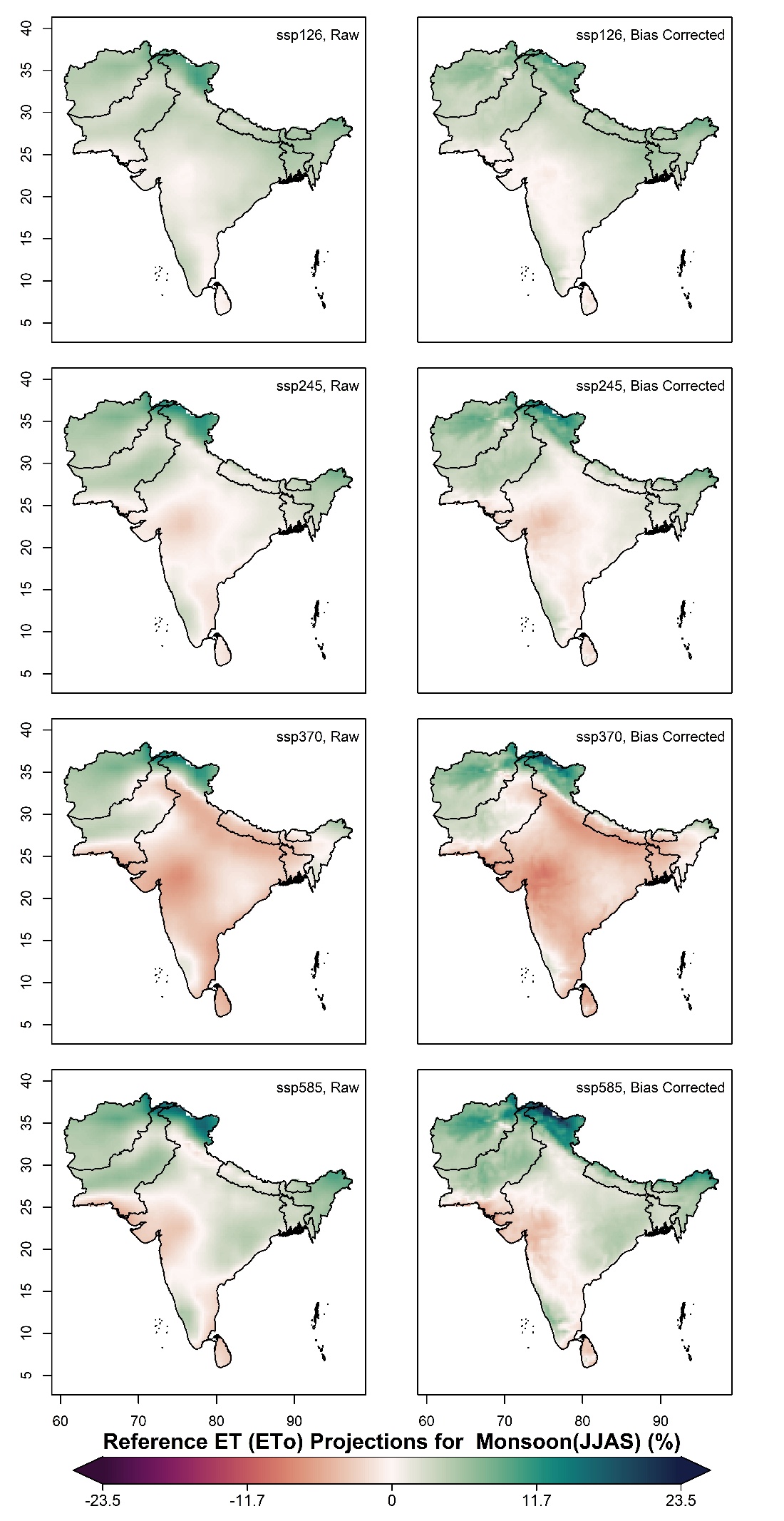


**Figure S 3:** same as Fig S2, but for monsoon (JJAS)


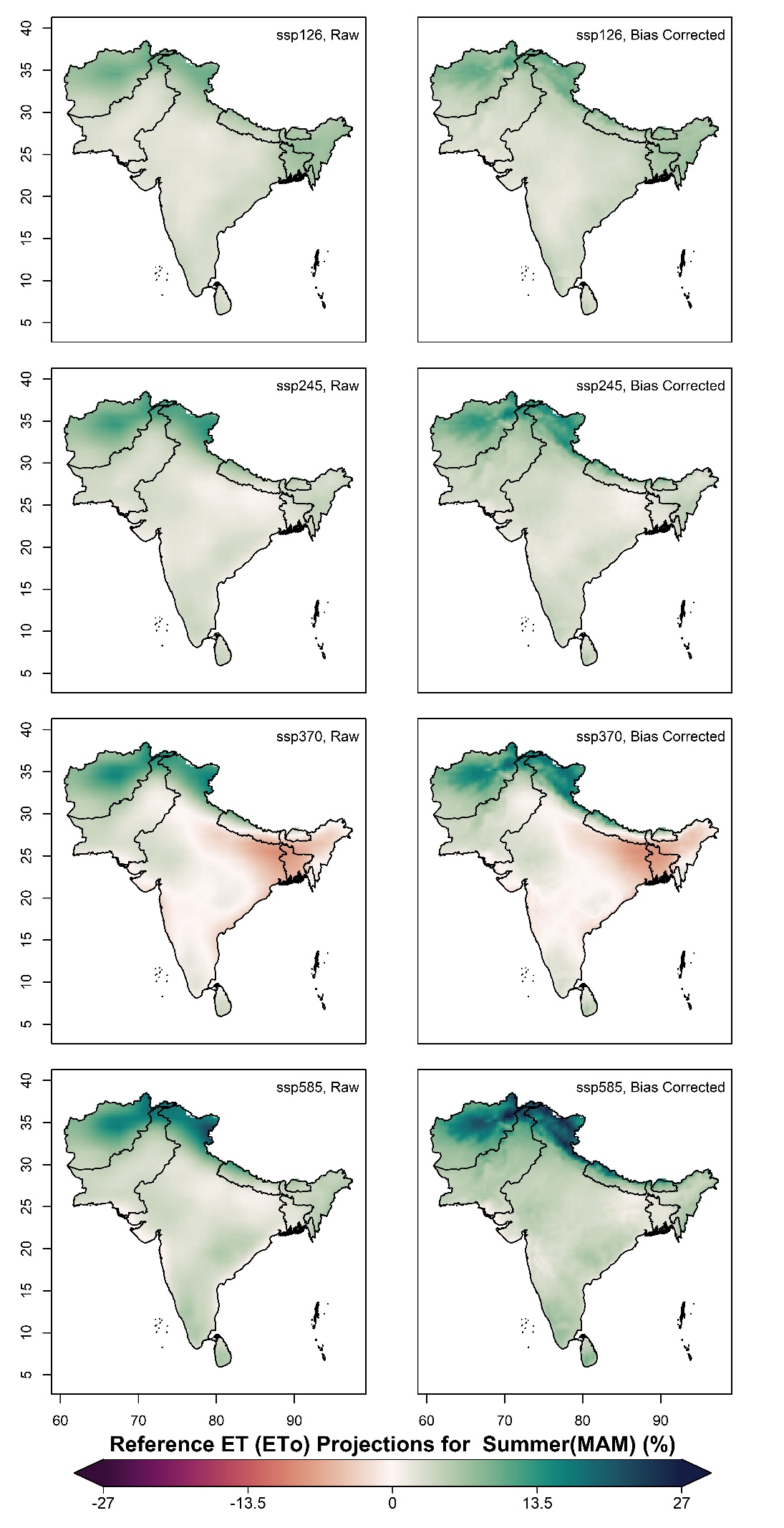


**Figure S 4 :** same as Fig S2, but for summer (MAM)


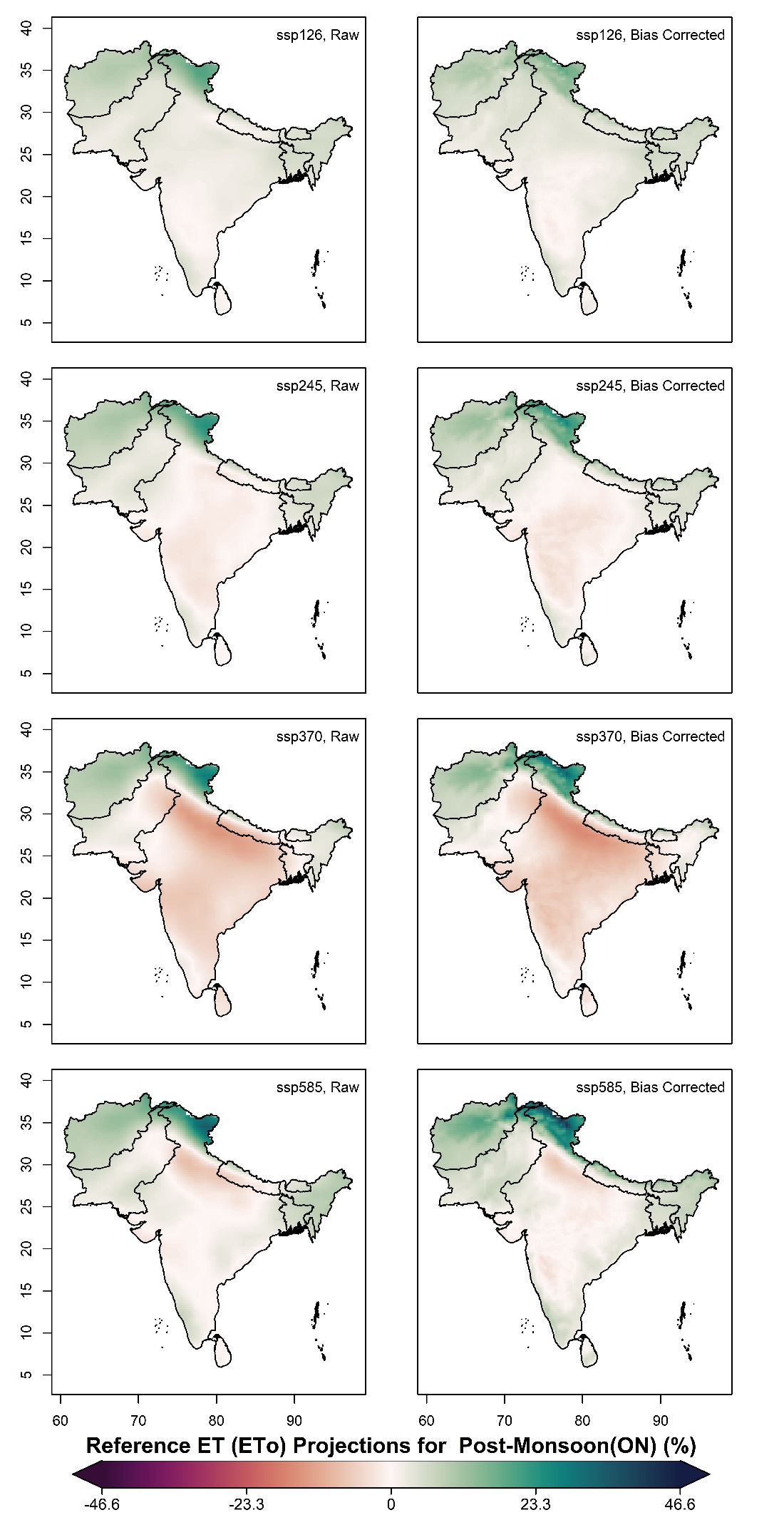


**Figure S5 :** same as Fig S2, but for post-monsoon (ON)

Table S2 : Country wise multi-model ensemble mean projected change in relative humidity (%). Historical mean is provided in %. The standard deviation(±1SD) represents the spread of the multi-model ensemble.

| **Country** | **Historic Mean (%)** | **ssp126** | | **ssp245** | | **ssp370** | | **ssp585** | |
| --- | --- | --- | --- | --- | --- | --- | --- | --- | --- |
|  |  | **Near** | **Far** | **Near** | **Far** | **Near** | **Far** | **Near** | **Far** |
| Afghanistan | 45.63 | -0.98 ± 0.60 | -1.66 ± 0.53 | -1.13 ± 0.61 | -1.96 ± 0.77 | -1.25 ± 0.75 | -1.65 ± 1.02 | -1.66 ± 0.77 | -2.30 ± 1.17 |
| Bangladesh | 76.86 | 0.40 ± 0.19 | -0.33 ± 0.20 | 1.48 ± 0.42 | 0.48 ± 0.28 | 2.22 ± 1.05 | 2.00 ± 1.10 | 1.09 ± 0.46 | 0.93 ± 0.44 |
| Bhutan | 80.85 | 0.61 ± 0.12 | 0.18 ± 0.14 | 0.91 ± 0.31 | 0.66 ± 0.15 | 1.29 ± 0.46 | 1.54 ± 0.41 | 0.84 ± 0.18 | 1.28 ± 0.13 |
| India | 62.23 | 1.25 ± 0.77 | 0.81 ± 0.85 | 1.72 ± 0.99 | 1.64 ± 1.28 | 2.66 ± 1.41 | 2.93 ± 1.69 | 1.78 ± 1.08 | 2.20 ± 1.58 |
| Nepal | 70.48 | 0.93 ± 0.41 | 0.31 ± 0.32 | 1.30 ± 0.86 | 0.95 ± 0.74 | 2.20 ± 1.26 | 2.22 ± 1.50 | 1.26 ± 0.92 | 1.54 ± 1.06 |
| Pakistan | 45.79 | 0.46 ± 0.52 | 0.02 ± 0.71 | 0.66 ± 0.67 | 0.35 ± 0.91 | 1.22 ± 0.95 | 1.77 ±1.33 | 0.73 ± 0.78 | 1.10 ± 1.25 |
| Sri Lanka | 78.38 | 0.59 ± 0.10 | 0.67 ± 0.14 | 0.75 ± 0.13 | 0.90 ± 0.15 | 0.90 ± 0.19 | 1.10 ± 0.29 | 0.72 ± 0.20 | 0.81 ± 0.28 |

Table S3: Country wise multi-model ensemble mean projected change in solar radiation (W/m^2^ ). Historical mean is provided in MJ m^-2^ d^-1^. The standard deviation(±1SD) represents the spread of the multi-model ensemble.

| **Country** | **Historic Mean** | **ssp126** | | **ssp245** | | **ssp370** | | **ssp585** | |
| --- | --- | --- | --- | --- | --- | --- | --- | --- | --- |
|  |  | **Near** | **Far** | **Near** | **Far** | **Near** | **Far** | **Near** | **Far** |
| Afghanistan | 247.53 | -0.07 ± 0.78 | 1.32 ± 0.80 | -1.63 ± 0.86 | -0.62 ± 0.95 | -3.84 ± 1.10 | -5.23 ± 1.52 | -1.44 ± 1.00 | -2.18 ± 1.26 |
| Bangladesh | 197.96 | -0.22 ± 0.37 | 5.36 ± 0.96 | -9.84 ± 1.64 | -0.98 ± 0.58 | -16.55 ± 2.74 | -14.31 ± 2.55 | -6.47 ± 1.02 | -2.15 ± 0.52 |
| Bhutan | 187.54 | -0.84 ± 0.65 | 3.50 ± 1.12 | -6.91 ± 0.91 | -1.10 ± 0.75 | -11.46 ± 1.44 | -10.81 ± 1.34 | -4.50 ± 0.70 | -2.24 ± 1.14 |
| India | 224.54 | -1.40 ± 1.10 | 2.86 ± 1.87 | -8.72 ± 2.39 | -2.99 ± 1.60 | -14.50 ± 3.70 | -14.09 ± 3.30 | -7.55 ± 2.16 | -5.06 ± 2.29 |
| Nepal | 216.91 | -1.42 ± 0.84 | 2.45 ± 1.83 | -8.30 ± 1.49 | -2.90 ± 0.94 | -13.64 ± 2.62 | -13.59 ± 1.93 | -6.45 ± 0.79 | -4.88 ± 1.48 |
| Pakistan | 245.41 | -1.10 ± 0.54 | 0.74 ± 1.07 | -4.58 ± 1.47 | -2.15 ± 1.06 | -9.02 ± 2.86 | -11.35 ± 3.17 | -4.25 ± 1.24 | -5.17 ± 1.60 |
| Sri Lanka | 231.75 | -1.83 ± 0.21 | 0.61 ± 0.52 | -5.11 ± 0.42 | -2.50 ± 0.26 | -8.67 ± 0.70 | -8.30 ± 0.68 | -4.65 ± 0.61 | -3.00 ± 0.36 |

Table S4: Country wise multi-model ensemble mean projected change in wind speed (ms^-1^). Historical mean is provided in ms^-1^. The standard deviation(±1SD) represents the spread of the multi-model ensemble.

| **Country** | **Historic Mean** | **ssp126** | | **ssp245** | | **ssp370** | | **ssp585** | |
| --- | --- | --- | --- | --- | --- | --- | --- | --- | --- |
|  |  | **Near** | **Far** | **Near** | **Far** | **Near** | **Far** | **Near** | **Far** |
| Afghanistan | 2.51 | -0.05 ± 0.02 | -0.04 ± 0.03 | -0.06 ± 0.02 | -0.06 ± 0.04 | -0.07 ± 0.03 | -0.09 ± 0.04 | -0.06 ± 0.03 | -0.08 ± 0.05 |
| Bangladesh | 2.38 | -0.03 ± 0.01 | -0.02 ± 0.02 | -0.04 ± 0.02 | -0.05 ± 0.03 | -0.04 ± 0.04 | -0.01 ± 0.05 | -0.02 ± 0.04 | 0.01 ± 0.05 |
| Bhutan | 1.07 | -0.04 ± 0.02 | -0.04 ± 0.02 | -0.05 ± 0.03 | -0.03 ± 0.04 | -0.06 ± 0.03 | -0.08 ± 0.05 | -0.10 ± 0.05 | -0.10 ± 0.07 |
| India | 2.42 | -0.03 ± 0.03 | -0.02 ± 0.04 | -0.03 ± 0.03 | -0.02 ± 0.04 | -0.05 ± 0.04 | -0.04 ± 0.05 | -0.02 ± 0.04 | 0.02 ± 0.07 |
| Nepal | 1.30 | -0.06 ± 0.01 | -0.06 ± 0.01 | -0.08 ± 0.01 | -0.09 ± 0.02 | -0.10 ± 0.02 | -0.13 v 0.02 | -0.10 ± 0.02 | -0.11 ± 0.03 |
| Pakistan | 2.67 | -0.01 ± 0.04 | 0.00 ± 0.05 | -0.02 ± 0.04 | -0.01 ± 0.06 | -0.04 ± 0.04 | -0.03 ± 0.07 | -0.02 ± 0.04 | 0.01 ± 0.10 |
| Sri Lanka | 3.65 | -0.11 ± 0.01 | -0.16 ± 0.02 | -0.10 ± 0.04 | -0.15 ± 0.06 | -0.15 ± 0.02 | -0.23 ± 0.03 | -0.07 ± 0.04 | -0.13 ± 0.06 |

Table S5: Country wise multi-model ensemble mean projected change in Temperature (K). Historical mean is provided in K. The standard deviation(±1SD) represents the spread of the multi-model ensemble.

| **Country** | **Historic Mean** | **ssp126** | | **ssp245** | | **ssp370** | | **ssp585** | |
| --- | --- | --- | --- | --- | --- | --- | --- | --- | --- |
|  |  | **Near** | **Far** | **Near** | **Far** | **Near** | **Far** | **Near** | **Far** |
| Afghanistan | 285.19 | 2.01 ± 0.08 | 2.26 ± 0.09 | 2.36 ± 0.08 | 3.37 ± 0.10 | 2.57 ± 0.10 | 4.50 ± 0.14 | 2.98 ± 0.10 | 5.58 ± 0.12 |
| Bangladesh | 298.18 | 1.21 ± 0.07 | 1.50 ± 0.10 | 1.23 ± 0.05 | 2.06 ± 0.12 | 1.23 ± 0.12 | 2.62 ± 0.13 | 1.65 ± 0.08 | 3.61 ± 0.17 |
| Bhutan | 282.58 | 1.44 ± 0.09 | 1.68 ± 0.08 | 1.61 ± 0.13 | 2.44 ± 0.13 | 1.71 ± 0.16 | 3.18 ± 0.21 | 2.04 ± 0.14 | 4.14 ± 0.18 |
| India | 295.42 | 1.35 ± 0.26 | 1.58 ± 0.26 | 1.50 ± 0.30 | 2.31 ± 0.37 | 1.51 ± 0.34 | 3.03 ± 0.50 | 1.94 ± 0.35 | 4.09 ± 0.56 |
| Nepal | 285.10 | 1.52 ± 0.14 | 1.78 ± 0.15 | 1.69 ± 0.23 | 2.55 ± 0.23 | 1.73 ± 0.30 | 3.32 ± 0.42 | 2.15 ± 0.25 | 4.41 ± 0.36 |
| Pakistan | 295.34 | 1.72 ± 0.15 | 1.95 ± 0.17 | 1.99 ± 0.20 | 2.95 ± 0.25 | 2.08 ± 0.24 | 3.84 ± 0.36 | 2.54 ± 0.23 | 4.97 ± 0.39 |
| Sri Lanka | 299.68 | 1.02 ± 0.02 | 1.15 ± 0.02 | 1.17 ± 0.02 | 1.77 ± 0.02 | 1.29 ±0.03 | 2.39 ± 0.03 | 1.53 ± 0.03 | 3.09 ± 0.04 |

Table S6 : Country wise multi-model ensemble mean projected change in ETo (mm). Historical mean is provided in mm. The standard deviation(±1SD) represents the spread of the multi-model ensemble.

| **Country** | **Historic Mean** | **ssp126** | | **ssp245** | | **ssp370** | | **ssp585** | |
| --- | --- | --- | --- | --- | --- | --- | --- | --- | --- |
|  |  | **Near** | **Far** | **Near** | **Far** | **Near** | **Far** | **Near** | **Far** |
| Afghanistan | 1511.45 | 63.55 ± 16.45 | 85.32 ± 21.80 | 67.41 ± 16.66 | 102.56 ± 21.93 | 61.77 ± 18.60 | 103.73 ± 22.05 | 86.57 ± 21.23 | 137.99 ± 21.38 |
| Bangladesh | 1291.84 | 19.33 ± 3.88 | 65.60 ± 8.38 | -41.00 ± 12.62 | 30.77 ± 8.79 | -85.38 ± 28.94 | -46.45 ± 28.97 | -13.45 ± 11.36 | 43.86 ± 13.86 |
| Bhutan | 749.26 | 21.27 ± 4.00 | 44.42 ± 4.86 | 0.54 ± 15.87 | 38.14 ± 5.46 | -16.67 ± 24.05 | 8.62 ± 24.33 | 17.03 ± 12.89 | 58.36 ± 8.96 |
| India | 1517.48 | 12.92 ± 14.56 | 44.70 ± 15.76 | -19.83 ± 26.92 | 26.45 ± 21.11 | -59.21 ± 41.78 | -26.95 ± 41.13 | -6.08 ± 25.55 | 46.50 ± 26.14 |
| Nepal | 979.57 | 20.91 ± 6.53 | 47.19 ± 5.84 | -7.16 ± 26.93 | 35.94 ± 11.27 | -35.31 ± 44.83 | -4.44 ± 43.78 | 8.37 ± 22.38 | 59.07 ± 15.12 |
| Pakistan | 1847.89 | 40.88 ± 11.26 | 62.91 ± 14.36 | 29.99 ± 18.66 | 70.25 ± 20.49 | 2.27 ± 29.97 | 30.16 ± 40.30 | 43.49 ± 20.94 | 90.03 ± 34.01 |
| Sri Lanka | 1520.70 | 5.98 ± 8.42 | 20.02 ± 7.48 | -9.08 ± 12.32 | 13.59 ± 14.61 | -28.60 ± 17.79 | -14.60 ± 27.80 | 1.32 ± 16.89 | 23.69 ± 30.34 |
